# Carboxypeptidases for food processing: structural characteristics, expression optimization, and advances in applications

**DOI:** 10.3389/fnut.2026.1915795

**Published:** 2026-09-21

**Authors:** Yitong Du, Junqi Liu, Xinyu Zhu, Ting Yu, Xiyan Wang

**Affiliations:** 1 College of Food Science and Engineering, Jilin Agricultural University, Changchun, China; 2 Department of Nutrition, The Second Hospital of Jilin University, Changchun, China

**Keywords:** carboxypeptidases, catalytic mechanisms, debittering, heterologous expression, peptide preparation

## Abstract

Carboxypeptidases, a class of exopeptidases that specifically cleave C-terminal amino acid residues from peptide chains, are extensively utilized for food component modification and quality enhancement. This review focuses on the three-dimensional structural characteristics and active-site catalytic mechanisms of serine carboxypeptidases, metallocarboxypeptidases, and cysteine carboxypeptidases and describes the construction and optimization strategies for heterologous expression systems, with particular emphasis on *Escherichia coli* (*E. coli*), *Bacillus subtilis*, and *Pichia pastoris*. Furthermore, the use and current research status of carboxypeptidases in food processing are comprehensively explored, including the debittering of protein hydrolysates, the targeted preparation of high-Fischer-ratio (high-F-ratio) oligopeptides, the quality enhancement of fermented foods, and the green biological detoxification of ochratoxin A (OTA). Further research can focus on enhancing the substrate specificity and catalytic efficiency of carboxypeptidases through molecular modification. This review aims to provide a theoretical foundation for the broader application of carboxypeptidases in green food manufacturing and quality improvement.

## 1 Introduction

Carboxypeptidases are a class of exopeptidases characterized by their ability to sequentially cleave amino acid residues from the C-terminus of a peptide chain, thereby releasing free amino acids. Based on the structural distinctiveness of their catalytic active site, carboxypeptidases are primarily classified into three major groups: serine carboxypeptidases, metallocarboxypeptidases, and cysteine carboxypeptidases (1, 2). Consequently, due to variations in their enzymatic architecture, active sites, and biological origins, these carboxypeptidases exhibit extensive diversity in their physiological functions and catalytic properties. For instance, pancreatic carboxypeptidases A and B facilitate digestive processes (3), and carboxypeptidase M contributes to the processing of peptides selectively (4).

Carboxypeptidases are widely found in higher plants, animals, and microorganisms. Among these, microbial carboxypeptidases, due to their wide availability and ease of cultivation and extraction, have become a key research focus as food-processing enzymes. However, wild strains often produce low levels of these enzyme and exhibit complex expression systems. Therefore, researchers have used *Escherichia coli* (*E. coli*), *Pichia pastoris*, and *Bacillus subtilis* as hosts for a heterologous expression of carboxypeptidases (5).

In food processing, carboxypeptidases cleave C-terminal amino acid residues from proteins, generating small peptides and amino acids with specific flavor and functional properties. They are therefore used to improve the flavor, texture, and nutritional value of food products, as exemplified by the preparation of high-Fischer-ratio (high-F-ratio) oligopeptides (6), the removal of ochratoxin A (OTA) (7) and the debittering agents (2, 8).

This review discusses the structure and classification of carboxypeptidases, the development of their expression systems, and their key applications in food processing. Furthermore, it provides novel perspectives on future research directions, with a particular focus on enhancing enzymatic properties through molecular modification strategies, such as semi-rational design and directed evolution, to address challenges in current applications. The present review provides a theoretical foundation for the in-depth development and high-value utilization of carboxypeptidases in the food industry.

## 2 Structure and catalytic mechanisms of carboxypeptidases

Based on the characteristics of their active sites, carboxypeptidases are classified into three major groups: Serine carboxypeptidases, metallocarboxypeptidases, and cysteine carboxypeptidases (9). Although carboxypeptidases from different families exhibit extremely low sequence homology and considerable sequence divergence, they form similar active-site architectures through specific spatial folding. The spatial conformation of carboxypeptidases is characterized by a relatively compact polar backbone and a low-density hydrophobic region at the catalytic center, resembling an irregular honeycomb-like structure, in which eight helix regions, eight β-strands, and approximately 45 turns collectively account for 90% of the total residues in the protease. By catalyzing the hydrolysis of C-terminal peptide bonds in peptide chains or proteins, carboxypeptidases participate in physiological processes such as the degradation of specific substrate molecules and the functional maturation of other molecules.

### 2.1 Serine carboxypeptidases

Serine carboxypeptidases are a class of carboxypeptidases that contain a serine residue in their active-site region and are also known as acid carboxypeptidases. They are the most widely distributed and extensively applied carboxypeptidases. As eukaryotic proteases, they belong to family S10 of clan SC and typically have subunit molecular masses of 40–75 kDa. They are widely distributed in fungal, higher plant, and animal tissues (10). Under acidic conditions, serine carboxypeptidases exhibit exopeptidase, esterase, and deamidase activities, allowing them to participate in the processing, modification, and degradation of peptides and proteins. Structurally similar to serine proteases, serine carboxypeptidases harbor an active site that contains a catalytic unit sequentially composed of Ser, Asp., and His, in which Ser acts as the nucleophilic site, Asp serves as the electrophile, and His functions as the general base (11). In the active-site region, the conserved sequence G-X-S-X surrounds the serine, forming a charge transfer system within the tertiary structure of serine carboxypeptidases that enables substrate binding and facilitates the nucleophilic reaction. Serine carboxypeptidases also possess an oxyanion hole, a common feature of peptidases, which facilitates the stabilization of the enzyme–substrate complex (12–14). The catalytic center of carboxypeptidase Y consists of the catalytic triad Ser146-His397-Asp338 (Figure 1A). During catalysis, Ser146 nucleophilically attacks the carbonyl carbon of the peptide bond, forming an acyl-enzyme intermediate. Subsequently, a water molecule is activated by His397, which hydrolyzes the acyl-enzyme intermediate, thereby completing the catalytic reaction (15). It is widely used for bioactive peptide preparation and amino acid sequence analysis and is also the most commonly used enzyme tool for C-terminal analysis of proteins and polypeptides (10). Some serine carboxypeptidases are widely involved in protein maturation processes in animals. For example, carboxypeptidase D participates in post-translational modifications within subcellular compartments such as the endoplasmic reticulum.

**Figure 1:**
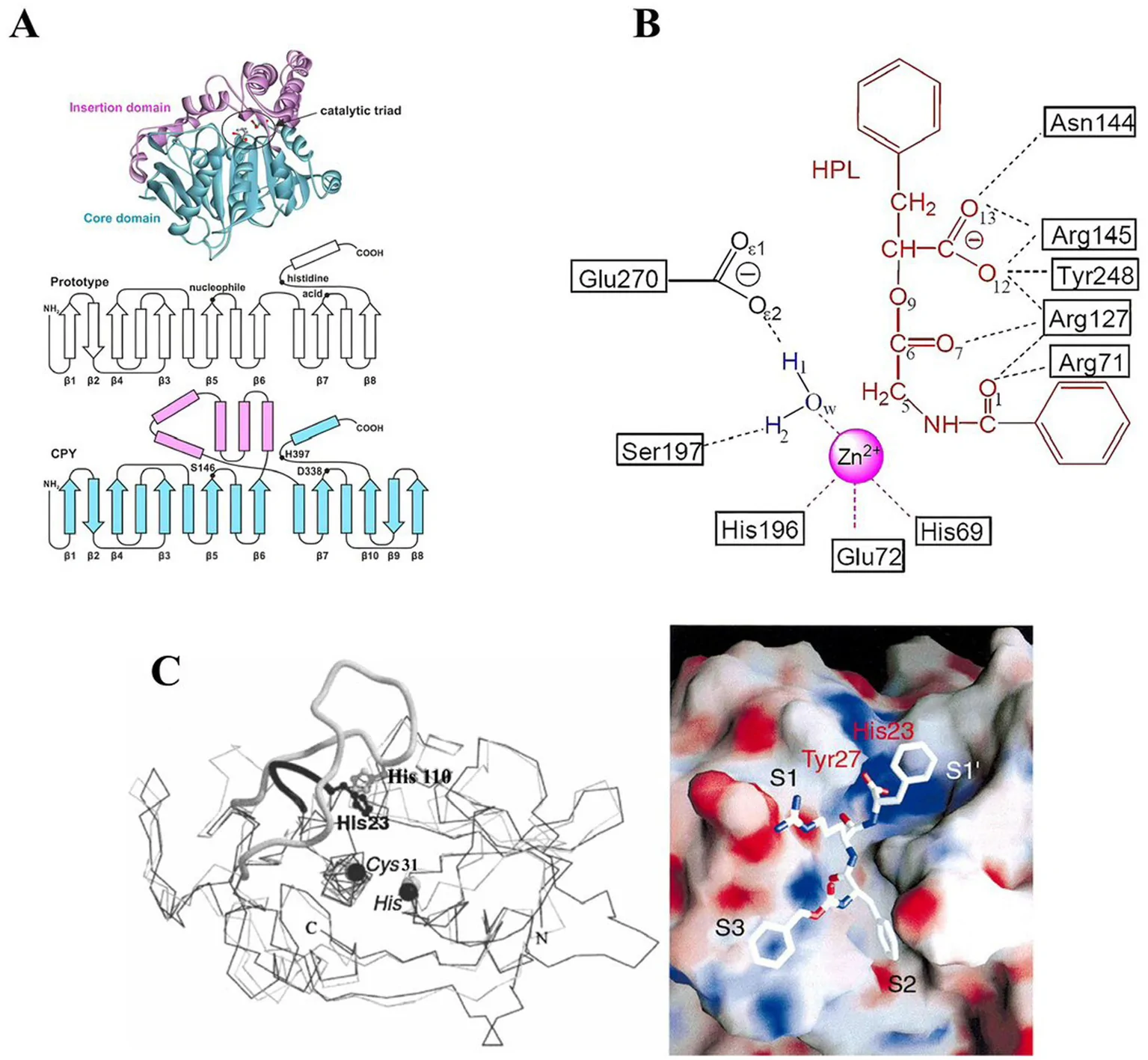
Catalytic mechanisms of **(A)** serine carboxypeptidases. Adapted from Figure 1 in (15), under the terms of the CC BY 4.0 International License (https://creativecommons.org/licenses/by/4.0/). **(B)** metallocarboxypeptidases. Adapted from SCHEME 2 in (20), with permission from the publisher. and **(C)** cysteine carboxypeptidases. Adapted from Figure 1 and Figure 3 in (23), with permission from the publisher.

### 2.2 Metallocarboxypeptidases

The three-dimensional structure of metallocarboxypeptidases adopts a typical α/β hydrolase fold, with the active site located in a cavity that contains Zn^2+^ (16). The most thoroughly studied metallocarboxypeptidases belong to the M14 family, which includes the initially discovered carboxypeptidases A and B. These two carboxypeptidases exhibit a high degree of structural similarity, with active sites containing Zn^2+^ coordinated by two His residues and one Glu residue, forming the characteristic “HEXXH” motif (17). The active site of carboxypeptidase A (PDB ID: 1AYE) comprises one catalytic site and three binding sites. His69, Glu72, and His196 constitute the three protein ligands coordinating the catalytic Zn^2^ ion, while the coordination sphere is completed by a loosely bound water molecule. Flanking the zinc–water axis, Arg127, Arg145, Asn144, and Glu270 participate directly in the catalytic process (18). During catalysis, the Zn^2+^ ion simultaneously polarizes both the coordinated water molecule and the substrate carbonyl oxygen, thereby enhancing the nucleophilicity of the water molecule and the electrophilicity of the carbonyl oxygen (19). As shown in Figure 1B, when the substrate binds, Glu270 acts as a general base to deprotonate the Zn^2+^-bound water molecule, generating a hydroxide ion that attacks the scissile peptide bond, forming a tetrahedral intermediate. This negatively charged intermediate is stabilized by the Zn^2+^ ion and the oxyanion hole formed by Arg127 and Asn144. Subsequently, Glu270 serves as a general acid, protonating the remaining amine group and facilitating the breakdown of the tetrahedral intermediate, ultimately releasing the cleavage products and regenerating the active enzyme (20, 21).

As a class of extracellular enzymes, metallocarboxypeptidases are extensively utilized in protein digestion, as they exhibit high activity under neutral-to-weakly alkaline conditions. For example, Pinto et al. (22) used metallocarboxypeptidases to hydrolyze cheese whey proteins, resulting in a substantial reduction in the bitterness of the resulting protein hydrolysate. In addition, certain proteins exhibit high domain-level similarity to metallocarboxypeptidases but lack essential catalytic sites. These proteins are therefore classified as carboxypeptidase-like proteins that participate in various important physiological reactions *in vivo*.

### 2.3 Cysteine carboxypeptidases

Cysteine carboxypeptidases, whose catalytic domains contain residues such as Cys84, His233, and Asn254, are also known as cathepsin X or cathepsin Z. In cathepsin X, Cys31 acts as a catalytic nucleophile and attacks the carbonyl carbon of the scissile peptide bond, leading to the formation of a covalent acyl-enzyme intermediate. The active-site cleft facilitates substrate recognition and positions the C-terminal peptide bond for hydrolysis. A distinctive mini-loop containing His23 and Tyr27 is located adjacent to the substrate-binding region and contributes to the restricted accessibility of the S2′ subsite, thereby favoring carboxypeptidase activity over endopeptidase activity. Structural modeling of substrate binding further illustrates how the substrate is accommodated within the active-site cleft (Figure 1C). Consistent with this structural specialization, cathepsin X shows minimal or no activity toward substrates terminating in Pro and exhibits only weak endopeptidase activity (23, 24). The catalytic cycle involves the formation of a covalent acyl-enzyme intermediate. These enzymes are widely present in the cytosol of tissues such as the digestive tract, brain, eyes, heart, and liver of various animals, including cattle, common carp, house mice, and brown flounder (25). Currently, research on the active-site specificity of cysteine carboxypeptidases is limited; however, it is clear that they show no activity toward C-terminal Pro and that their endopeptidase activity is very weak.

## 3 Expression systems for carboxypeptidases

Since the majority of wild-type carboxypeptidases exhibit low expression levels, complex expression systems, or unstable enzyme activity, they are unable to meet the demands of industrial production. Therefore, heterologous expression strategies have been widely adopted to introduce the target gene into non-native host cells, leveraging the host’s own protein folding, modification, and secretion machinery to improve the yield and activity of the target protein. Currently, *E. coli*, *Pichia pastoris,* and *Bacillus subtilis* are commonly used as the primary hosts for the efficient expression of carboxypeptidases.

### 3.1 Escherichia coli

*E. coli* is the most widely used expression system for heterologous proteins due to its well-defined genetics, straightforward manipulation, and robust capacity for heterologous protein production (26). Consequently, significant research attention has been directed toward strategies for further enhancing expression efficiency. Microbial carboxypeptidase genes derived from *Geobacillus* sp. strain SBS-4S and *Bacillus subtilis* CW14 have been expressed in *E. coli* BL21, resulting in increased enzyme activity (27). Animal-derived carboxypeptidases focus more on utilizing their biological functions to achieve applications in the fields of biology and medicine. A full-length carboxypeptidase gene, CCP6, of mouse origin was successfully expressed in *Arctic Express* (DE3) with deglutaminylation activity (28). Similarly, the carboxypeptidase 302 gene derived from shrimp ponds was successfully expressed in *E. coli,* and the recombinant enzyme exhibited high affinity for folate (29). Although *E. coli* expression systems are extensively utilized, they nevertheless present several inherent limitations. For instance, proteins containing disulfide bonds or glycosylated modifications are difficult to express properly; proteins may fail to fold correctly and thus cannot form the three-dimensional structures required for enzymatic activity; and the inability of recombinant proteins to undergo post-translational modifications can lead to the formation of inclusions. To address these issues, researchers have used various strategies, such as promoter and codon optimization and inducible expression, to enhance the expression of recombinant proteins in *E. coli* expression systems.

### 3.2 Pichia pastoris

As a commonly utilized eukaryotic microbial expression system, *Pichia pastoris* possesses a diverse array of enzymes within its endoplasmic reticulum and Golgi apparatus that facilitate protein folding, disulfide bond formation, and glycosylation. These features constitute a comprehensive post-translational modification expression system capable of efficiently expressing heterologous carboxypeptidase genes. Currently, researchers have widely adopted the *Pichia pastoris* expression system for the heterologous expression of animal-derived carboxypeptidase genes, achieving a significant increase in yield. Expression levels of porcine carboxypeptidase B1 reached 250 mg·L^−1^ in *K. phaffii* GS115 (30) and 320 mg·L^−1^ in *K. phaffii* SMD1168 (31). Meanwhile, the serine carboxypeptidase CapA from *Aspergillus niger* was successfully secreted, with a crude specific activity of 209.3 U/mg, exhibiting excellent thermal and pH stability tailored for food and feed sectors (32). In addition, a high-level expression of human carboxypeptidase B1 (hCPB1) was achieved through a signal peptide screening strategy (33). Despite these individual scaling successes, a persistent bottleneck of traditional *Pichia pastoris* systems is their reliance on methanol induction. The intrinsic toxicity of methanol continues to pose regulatory and safety concerns for widespread applications in the food industry.

### 3.3 Bacillus subtilis

*Bacillus subtilis* is a generally recognized as safe (GRAS) strain featuring a rapid growth cycle and a robust capacity for extracellular protein secretion. Thus, it has become an established model system for heterologous protein expression in the manufacturing of model enzymes, industrial food enzymes, and therapeutic enzymes. Zhang et al. (34) expressed γ-glutamyl transpeptidase from *Bacillus alcalophilus* in *Bacillus subtilis*. Zong et al. (35) optimized the expression of a carboxypeptidase gene in *B. subtilis* using the DegSU QS system, leading to a 65.9% increase in extracellular carboxypeptidase activity. Furthermore, expression optimization strategies, including the synergistic optimization of promoters and signal peptides in *B. subtilis* WB600, resulted in a remarkable 3-fold increase in the expression of *Bacillus megaterium* carboxypeptidase (BmeCPM32), achieving a peak enzyme activity of 249.8 U/mL (36).

In fact, naturally occurring carboxypeptidases often encounter several inherent challenges when applied in fields such as food and pharmaceutical industries, including insufficient enzymatic activity, poor substrate specificity, and unstable enzymatic properties. Consequently, molecular modifications of wild-type carboxypeptidases are required to enhance their properties and broaden their scope of applications.

Among the three expression systems, *E. coli* is the most widely used due to its well-defined genetic background and high heterologous protein yield. However, its lack of post-translational modification machinery often leads to inclusion body formation, which restricts its application in the food sector. *P. pastoris* possesses robust eukaryotic modification capabilities and achieves high production yields, yet its reliance on methanol induction limits its utility in food industry applications. In contrast, *B. subtilis* serves as the preferred host for food-industrial production, although its expression efficiency still requires further optimization.

## 4 Application of carboxypeptidases in food processing

### 4.1 Removal of bitterness from protein hydrolysates

Peptides are hydrolyzed from proteins, which often exhibit multiple bioactivities and are easily digested and absorbed by the human body, thereby showing broad application prospects in the food industry. However, during enzymatic hydrolysis, exposure of hydrophobic amino acids often imparts a bitter taste to protein hydrolysates, reducing their sensory acceptability. Enzymatic hydrolysis is a promising debittering approach as it integrates protein hydrolysis and debittering into a one-step process. A prime example of this strategy is the debittering of soybean protein hydrolysates. Carboxypeptidase from *Aspergillus melleus*, *Actinomucor elegans,* and *Bacillus megaterium* effectively reduced the bitterness of soybean protein hydrolysates (37).

Notably, some studies have focused on modifying wild-type carboxypeptidases to enhance their catalytic efficiency and debittering efficacy. A semi-rationally engineered mutant of BmeCPM32-M2 exhibited a 2.9-fold increase in catalytic efficiency while simultaneously reducing the bitterness of soy hydrolysates by 83% and boosting umami intensity by 55% (36). Cheung et al. (38) demonstrated that treatment of whey protein hydrolysates with aminopeptidase and carboxypeptidase significantly reduced their bitterness while retaining their ACE-inhibitory activity *in vitro*.

### 4.2 Preparation of high-F-ratio oligopeptides

High-F-ratio oligopeptides refer to protein precursors or hydrolysates composed of 2–9 amino acid residues, in which the molar ratio of branched-chain amino acids to aromatic amino acids (the F ratio) exceeds 20. Due to their unique amino acid composition, high-F-ratio oligopeptides exhibit multiple physiological activities, including alleviating or treating liver diseases, fighting fatigue, and treating phenylketonuria. The enzymatic process for preparing high-F-value oligopeptides can be divided into two steps: First, endopeptidases hydrolyze the protein substrate to form soluble peptides; then, exopeptidases cleave off the aromatic amino acids, which are subsequently separated and removed. Carboxypeptidase A specifically hydrolyzes peptide bonds at the C-terminus of aromatic or bulky aliphatic amino acid residues while exhibiting no hydrolytic activity toward basic or proline residues (17). Therefore, this specificity can be exploited to efficiently produce high-F-value oligopeptides. Carboxypeptidase A immobilized on activated agarose gel was used as a tool enzyme to prepare high-F-value oligopeptides (39). Furthermore, enzyme engineering strategies, such as site-directed mutagenesis, targeting key active-site residues (S135G, Y160S, and Y206S) in an *Aspergillus niger* carboxypeptidase significantly enhanced its catalytic activity, representing an increase of up to 25.31% compared to the wild-type counterpart (6). Future research could focus on enhancing the hydrolytic substrate specificity of carboxypeptidase, thereby expanding its potential applications in the food processing industry.

### 4.3 Reduction of OTA

OTA is a carcinogenic mycotoxin that is widely found in cereals, fruit, wine, coffee, and other products. Carboxypeptidases from diverse biological sources have emerged as biodegradation methods for the enzymatic detoxification of OTA. For example, bovine pancreatic carboxypeptidase A and metallocarboxypeptidase purified from *Aspergillus niger* have demonstrated the ability to achieve complete OTA degradation (40, 41). Subsequently, a carboxypeptidase from the *Bacillus subtilis* CW14 genome was expressed in *Escherichia coli,* successfully reducing OTA levels by 45.0% (42).

### 4.4 Flavor enhancement during soy sauce brewing

Umami improvement through enzymatic hydrolysis is beneficial for flavor enhancement during soy sauce brewing, in which carboxypeptidases play an important role. Carboxypeptidase-mediated enzymatic hydrolysis primarily occurs through two pathways. The first involves utilizing carboxypeptidases for targeted enzymatic hydrolysis to prepare hydrolysates enriched in desired umami peptides, thereby elevating the overall flavor of soy sauce (43). Alternatively, a two-step hydrolysis strategy can be used to increase the relative content of umami dipeptides, such as Asn-Pro and Ala-His, in protein hydrolysates, reduce the bitterness of soy sauce with a slight decrease in saltiness, and alter its sweetness and sourness (44).

### 4.5 Preparation of food-derived bioactive peptides

The sequence and spatial conformation of bioactive peptides often play crucial roles in their biological activity, particularly the amino acid composition at the C- and N-termini. Therefore, to obtain target peptide fragments with a defined sequence, peptides are typically prepared using a combination of endonucleases and exonucleases. Carboxypeptidase Y is regarded as a useful tool for bioactive peptide preparation, including the synthesis of the pentapeptide Leu-enkephalin and ACE- inhibitory peptides. Shan et al. (45) constructed a recombinant carboxypeptidase Y and investigated its effects on the ACE-inhibitory activity and bitterness of whey protein hydrolysates. Li et al. (46) used sequential enzymatic hydrolysis of crude maize peptides using α-chymopapain and carboxypeptidase A to obtain maize peptides with reduced bitterness that are effective in repairing liver damage. *Brassica carinata* protein hydrolysates were obtained by hydrolysis with trypsin, chymotrypsin, and carboxypeptidase A, and showed antioxidant activity and inhibitory activity against angiotensin-converting enzyme (47). We have summarized recent advances in the application of carboxypeptidases in food processing (Figure 2).

**Figure 2:**
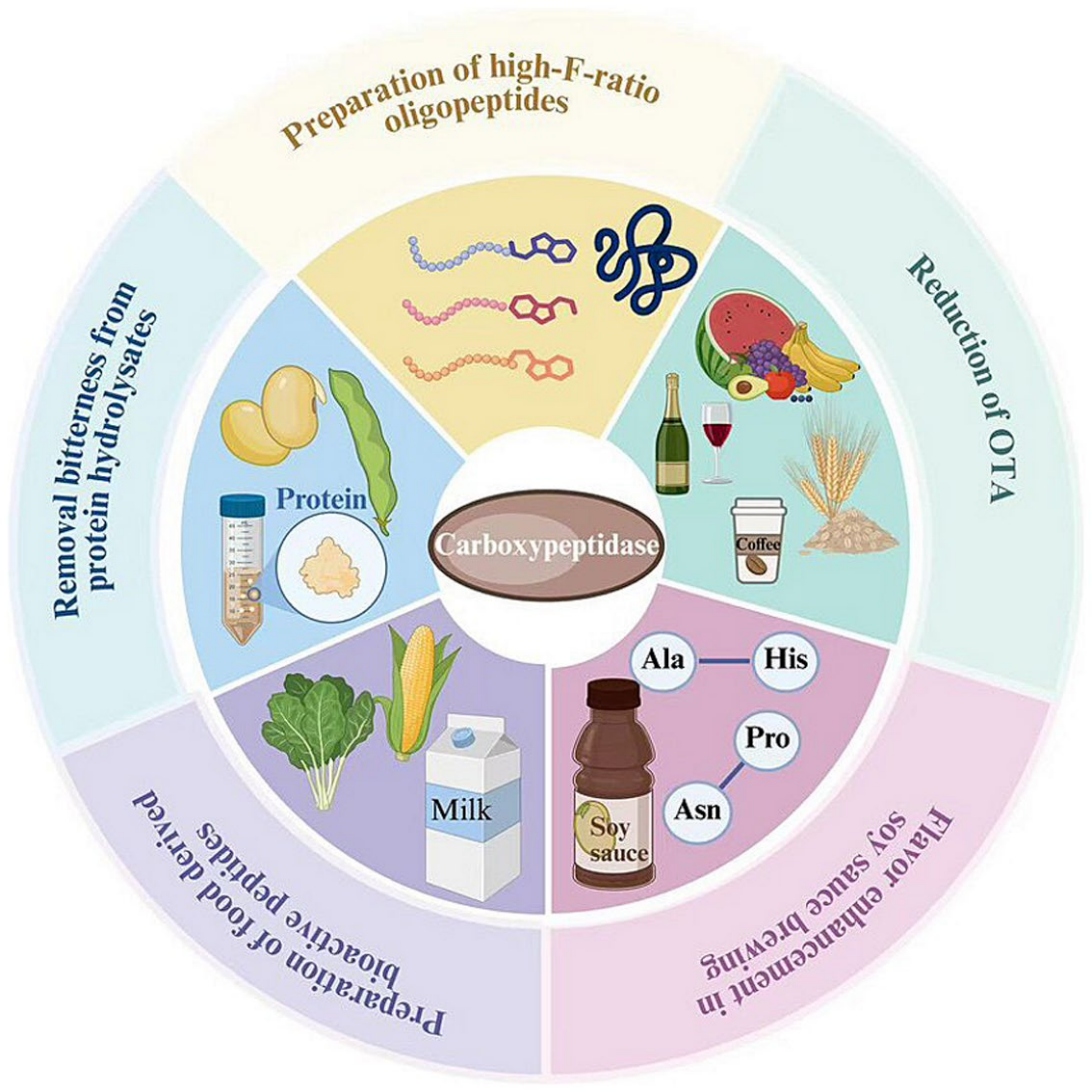
Application of carboxypeptidases in food processing (created with BioRender.com).

## 5 Conclusion

With the rapid development of enzyme preparations and the food industry, ensuring a safe and efficient application of these enzymes in food processing has become a key area of research. This review summarizes the structural characteristics, expression systems, and current applications of carboxypeptidases. It is widely used to improve the flavor of proteins, enhance the quality and efficiency of fermented foods, efficiently prepare food-derived bioactive peptides, and produce high-F-value oligopeptides. Nevertheless, several critical challenges remain to be addressed. First, the limited range and low efficiency of heterologous expression systems for carboxypeptidase genes restrict their practical applications. In addition, the substrate specificity of carboxypeptidases can lead to the loss of active components and reductions in product yield during the preparation of target bioactive peptides and debittering processes. Therefore, modern molecular biology techniques are required to elucidate and modify their substrate specificity, to further expand the practical applications of carboxypeptidases in the food industry.
